# MicroRNA-494 inhibits breast cancer progression by directly targeting PAK1

**DOI:** 10.1038/cddis.2016.440

**Published:** 2017-01-05

**Authors:** Meng-Na Zhan, Xiao-Ting Yu, Jun Tang, Ci-Xiang Zhou, Chen-Long Wang, Qian-Qian Yin, Xiu-Feng Gong, Ming He, Jian-Rong He, Guo-Qiang Chen, Qian Zhao

**Affiliations:** 1 Key Laboratory of Cell Differentiation and Apoptosis of National Ministry of Education, Department of Pathophysiology and Rui-Jin Hospital, Shanghai Jiao Tong University School of Medicine (SJTU-SM), Shanghai, China; 2 Department of Pathology, Tong-Ji Hospital Affiliated to Tong-Ji University School of Medicine, Shanghai, China; 3 Institute of Health Sciences, Shanghai Jiao Tong University School of Medicine (SJTU-SM) & Shanghai Institutes for Biological Sciences (SIBS), Chinese Academy of Sciences (CAS), Shanghai, China; 4 Department of General Surgery, Rui-Jin Hospital, Shanghai Jiao Tong University School of Medicine, Shanghai, China

## Abstract

MicroRNA (miRNA) is involved in the progression and metastasis of diverse human cancers, including breast cancer, as strong evidence has been found that miRNAs can act as oncogenes or tumor suppressor genes. Here, we show that miR-494 is decreased in human breast cancer specimens and breast cancer cell lines. Ectopic expression of miR-494 in basal-like breast cancer cell lines MDA-MB-231-LUC-D2H3LN and BT-549 inhibits clonogenic ability and metastasis-relevant traits *in vitro*. Moreover, ectopic expression of miR-494 suppresses neoplasm initiation as well as pulmonary metastasis *in vivo*. Further studies have identified PAK1, as a direct target gene of miR-494, contributes to the functions of miR-494. Remarkably, the expression of PAK1 is inversely correlated with the level of miR-494 in human breast cancer samples. Furthermore, re-expression of PAK1 partially reverses miR-494-mediated proliferative and clonogenic inhibition as well as migration and invasion suppression in breast cancer cells. Taken together, these findings highlight an important role for miR-494 in the regulation of progression and metastatic potential of breast cancer and suggest a potential application of miR-494 in breast cancer treatment.

Breast cancer is one of the most common female malignancy in the world. According to GLOBOCAN, in 2012 an estimated 1.67 million women were diagnosed with breast cancer and there were 6.3 million women alive who had been diagnosed with breast cancer in the previous 5 years.^1^ Meanwhile, the aging of current global population means that nearly 50% more women will develop and die from breast cancer in 2020 than 2002.^2^ Breast cancer is a highly heterogeneous disease encompassing a wide variety of pathological entities and a range of clinical behavior. And these features are underpinned by genetic alteration which cause cellular processes changes.^3, 4^ As such, the molecular mechanisms of the progression of breast cancer are still not clear, bringing a huge challenge in diagnosis and treatment. Recent years, the hallmarks of cancer have become highly influential in breast cancer research. The six hallmarks of cancer are distinctive and complementary capabilities that enable tumor growth and metastatic dissemination. Sustaining proliferative signaling is one of the biological capabilities acquired in the process of breast cancer that promotes tumor growth.^5^ And metastasis is considered to be poor prognosis in uterine cancer,^6^ prostate cancer^7^ and nearly all types of cancers. The multistep process of invasion and metastasis often termed the invasion-metastasis cascade, which begin with local invasion, then intravasation by cancer cells through the lymphatic and hematogenous systems, followed by escape of cancer cells from the lumina of such vessels into the parenchyma of distant tissue, the formation of small nodules of cancer cells, and finally the growth of micro metastasis lesions into macroscopic tumors.^8^

MicroRNAs (miRNAs) are endogenous ~23nt RNAs that play important gene regulatory roles in mammal by pairing to mRNAs of protein-coding genes to direct their posttranscriptional repression.^9^ Owing to imperfect complementary between miRNAs and their targets, each miRNA may possess >100 targets. This makes them powerful genes due to their ability to modulate 30% of proteins.^10^ Hence, it is not surprising that dysregulation of miRNAs is frequently implicated in many disease, including variety of cancers. Breast cancer is one of this kind of cancer.^11, 12, 13, 14^ Previous reports have elaborated that miRNAs are involved in various essential cellular processes of breast cancer, such as proliferation,^15^ apopotosis,^16^ stem-cell renewal,^17^ EMT^18^ and metastasis.^19^ In our previous study, we demonstrate that miR-124 has an important role in breast cancer invasion-metastasis cascade by targeting EMT regulator Slug^20^ and miR-630 suppresses breast cancer progression by targeting metadherin.^21^

MiR-494, first reported to be highly expressed in retinoblastoma,^22^ has been found in various types of human cancers. In the majority of cancers, miR-494 functions as a tumor suppressor gene. Meanwhile, conflicting reports indicated that the elevation of miR-494 has an oncogenic role in the progression of several kinds of cancers including non–small-cell lung cancer and colorectal cancer.^23, 24^ However, the function of miR-494 in breast cancer progression, especially its role in human breast cancer patients and in breast cancer mouse model, as well as the molecular mechanisms by which miR-494 exerts its functions and modulates the malignant phenotypes of breast cancer cells, has not been fully understood.

In this study, we give a clear interpretation that miR-494, which is downregulated in breast cancer tissue, suppresses tumor growth *in vitro* and *in vivo*. Further studies show that miR-494 inhibits proliferation, colony formation, migration and invasion of breast cancer cells *in vitro*, as well as tumorigenesis and lung metastasis *in vivo*. Furthermore, PAK1, which acts as an oncogene in breast cancer by activating MAPK signal pathway and remolding cytoskeletal, is demonstrated to be a direct target gene of miR-494. Thus our findings provides valuable clues toward understanding the mechanisms of human breast cancer initiation and progression and presents an opportunity to develop more effective clinical therapies in the future.

## Results

### Downregulation of miR-494 in breast cancer

**Figure 1:**
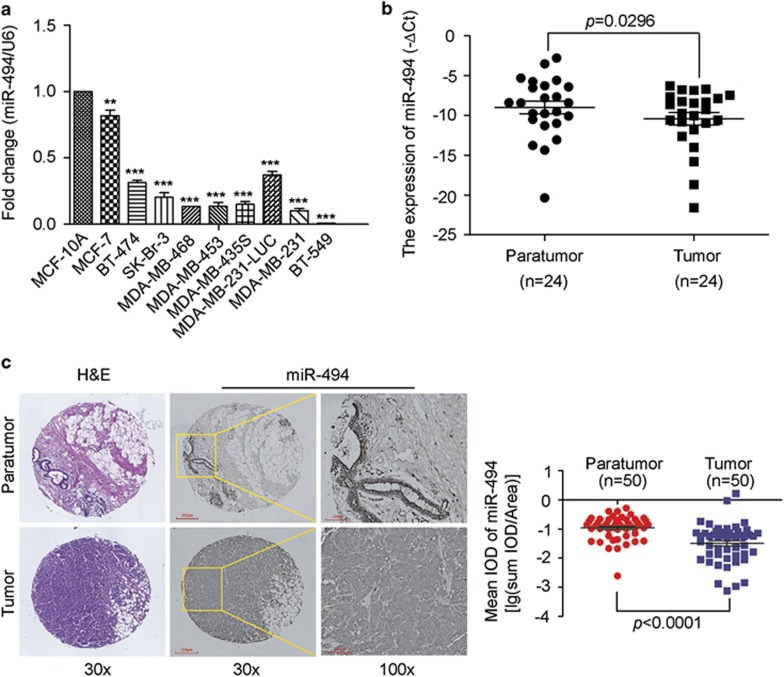
Expression of miR-494 in breast cancer cell lines and specimens. (**a**) Quantitative real-time PCR analysis of miR-494 expression in MCF-10A and nine breast cancer cell lines. The fold changes of relative expression of miR-494 versus that of MCF-10A are represented in the vertical axis. Experiments were performed three times. (**b**) Comparison of miR-494 abundance in 24 paired tumor and adjacent non-tumor tissues. The relative expression of miR-494 normalized to the internal control U6 is shown (*P*=0.0296, independent test). (**c**) *In situ* hybridization of miR-494 in breast cancer TMA (50 paired tumor and adjacent non-tumor tissues). The right is static map. Data are presented as mean±S.D. The symbols ** and *** denote significant statistical difference of *P*<0.01 and *P*<0.001 respectively by a two-tailed Student's *t*-test

To investigate the roles of miR-494 in breast cancer initiation and progression, we firstly detected the expression level of miR-494 in nine human breast cancer cell lines by real-time qRT-PCR. The result showed that the expression of miR-494 in all nine breast cancer cell lines were significantly reduced at different degrees compared with MCF-10A, an immortalized breast epithelial cell line (Figure 1a). Subsequently, the expression of miR-494 in clinical breast cancer tissues were also evaluated by qRT-PCR. From the result we can see miR-494 showed a lower expression in breast cancer tissues compared with the adjacent non-neoplastic tissues (Figure 1b). However, the expression stratification of miR-494 was independent of clinical pathological characteristics such as nodal status, ER status, HER2 status and so on (Supplementary Tables 1 and 2). To further describe miR-494 expression levels within mammary epithelial cells in the context of mammary architecture, a breast tissue microarray that contained 50 breast cancer and paired non-neoplastic specimens were used for further analysis. We applied *in situ* hybridization with a 3′ and 5′ DIG-labeled miR-494 probe on this TMA. And the data of tumor IODs and areas were collected by Image-Pro Plus 6.0 (IPP). In accordance with the qRT-PCR analysis, strong positive expression of miR-494 was observed in adjacent normal breast tissue whereas very weak positive expression of miR-494 in infiltrating ductal carcinoma (Figure 1c). Moreover, high expression of miR-494 was significantly associated with E-cadherin expression, but not with other clinical parameters (Supplementary Tables 3 and 4). These results indicated that the reduced miR-494 expression was a frequent event in human breast cancer cells and tissues, which may be involved in breast carcinoma progression.

### Exogenetic overexpression of MiR-494 suppresses clonogenic ability and metastasis-relevant traits *in vitro*

**Figure 2:**
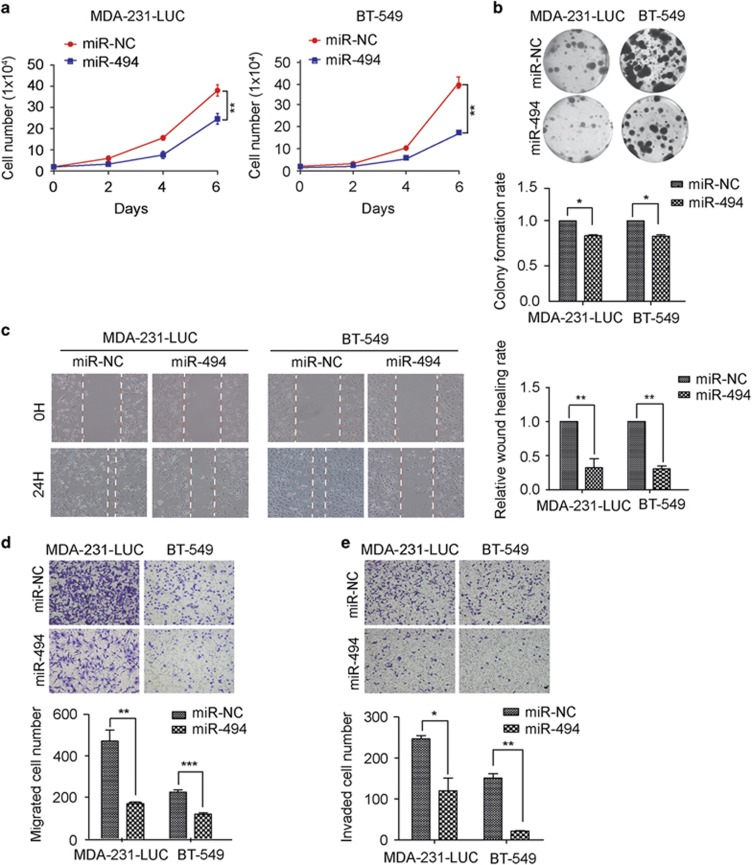
Overexpression of miR-494 suppresses proliferation, colony-forming, migration and invasion of breast cancer cells *in vitro.* (**a**) Growth curves of MDA-231-LUC and BT-549 cells after transfection of miR-NC or miR-494 mimics for 48 h. (**b**) Representative images of colony-forming ability in MDA-231-LUC and BT-549 cells after transiently transfection. (**c**) Wound healing assay of MDA-231-LUC and BT-549 after transfection of miR-NC or miR-494 mimics. Representative images depicting the beginning (*t*=0 h) and the end (*t*=24 h) of the recording are shown. (**d**) Transwell migration assay measuring MDA-231-LUC and BT-549 transfected with miR-NC or miR-494 mimics, respectively. Cell migration was analyzed 18 h after seeding in Transwells. (**e**) Invasion ability of MDA-231-LUC and BT-549 transfected with miR-NC or miR-494 mimics. Cell invasion was analyzed 18 h after seeding in Transwells. Data are representative of three independent experiments. Bar graphs show means of three experiments±S.D. The symbols *, ** and *** represent great significant difference (*P*<0.05, *P*<0.01 and *P*<0.001) by two-tailed Student's *t*-test

As MDA-MB-231-LUC-D3H2LN (hereafter referred to as MDA-231-LUC) and BT-549 cells have a lower expression level of miR-494 among the cultured breast cancer cells evaluated, we performed further functional experiments mainly in these two cell lines. Compared with normal cells, cancer cells could acquire the capability to sustain proliferative signaling. As miR-494 is downregulated in breast cancer tissue and breast cancer cell lines, we infer miR-494 would inhibit cancer cell proliferation. To explore this assumption, we transiently transfected MDA-231-LUC and BT-549 cells with miR-NC or miR-494 mimics respectively. The ectopic expression of miR-494 significantly suppressed cell proliferation in both MDA-231-LUC and BT-549 cells (Figure 2a). To further confirm the effects of miR-494 in MDA-231-LUC and BT-549 cells, colony-forming assay was performed to investigate the changes in propagation ability. And the results showed that the colony number of miR-494 overexpressed cells was lower than that of the control group (Figure 2b). Considering tumor invasion and metastasis are common features of the most aggressive and lethal tumors, we detected the effects of miR-494 overexpression on metastasis-relevant traits *in vitro*. As expected, in wound healing and Transwell migration assays, MDA-231-LUC and BT-549 cells overexpressed miR-494 displayed a significant depression in migration compared with miR-NC group (Figures 2c and d). Meanwhile, ectopic expression of miR-494 in MDA-231-LUC and BT-549 cells also inhibited the invasion ability assessed by Matrigel invasion assays as well (Figure 2e). These results indicated that miR-494 ectopic expression in MDA-231-LUC and BT-549 cells can strikingly decrease the ability of proliferation, clonogenicity and motility *in vitro.*

### MiR-494 suppresses tumorigenesis as well as tumor invasion *in vivo*

**Figure 3:**
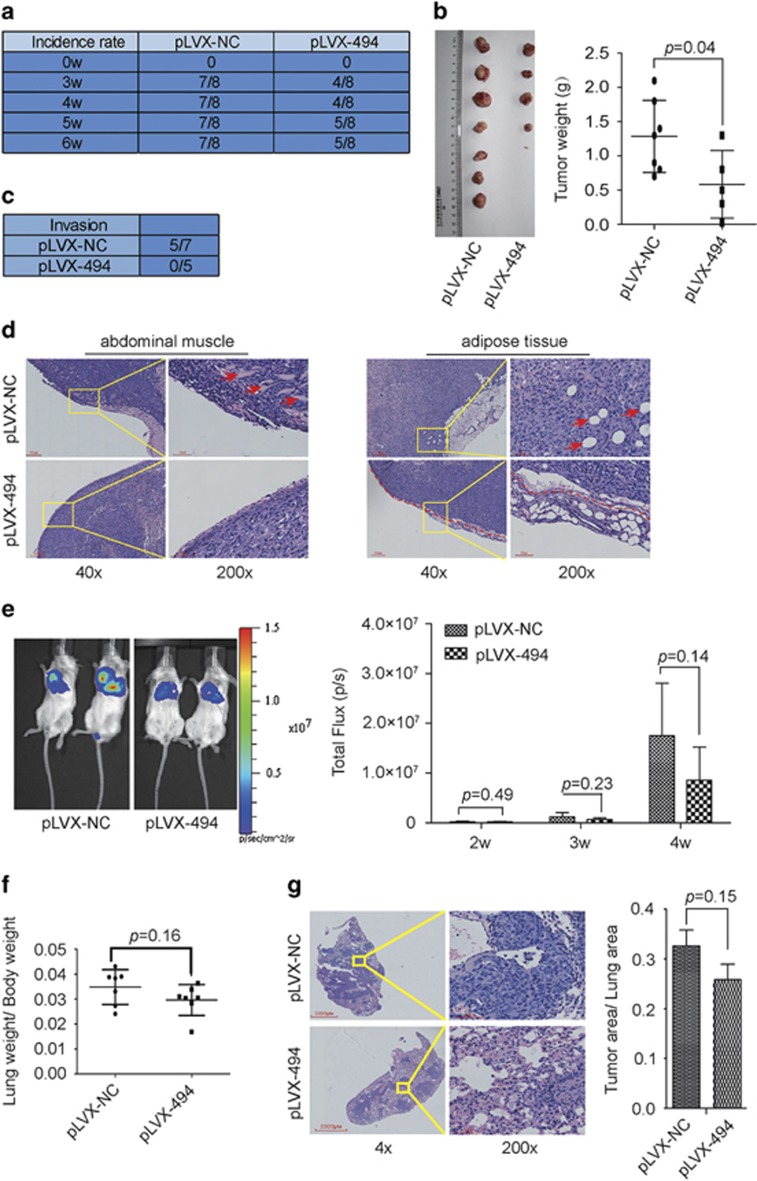
MiR-494 suppresses tumorigenesis and metastasis *in vivo*. (**a**) The incidence rate of tumors in pLVX-NC group and pLVX-494 group, 3 weeks after breast fat pad injection. (**b**) Tumors are separated from the indicated mice and weighted after 30 days post injection. (**c**) Statistics shows the incidence rate of tumor invading into peritoneal in pLVX-NC group and pLVX-494 group. (**d**) H&E staining of carcinoma and the tumor boundary of pLVX-NC group and pLVX-494 group (red arrows on the left panel indicating abdominal muscle and on the right panel indicating adipose tissue, and the dot line on the right is the boundary between tumor and adipose tissue). (**e**) *In vivo* IVIS luciferase images of lung metastasis are monitored using bioluminescent imaging. Representative lung metastasis burden of xenografted animals on second, third and fourth weeks after injected with pLVX-NC cells (*n*=7) and pLVX-494 cells (*n*=7). The right panel is the static column. (**f**) Lung weight with metastasis of pLVX-NC group and pLVX-494 group. (**g**) H&E staining analysis of tumor metastatic area. The right panel is the static column. Data are presented as mean±S.D.

Given that miR-494 inhibits cell growth, colony forming and motility *in vitro*, we constructed MDA-231-LUC cell line stably expressing miR-494 for *in vivo* assays. MiR-494 was cloned into pLVX-IRES-ZSGreen vector (Supplementary Figure 1A). qRT-PCR analysis showed the cells infected with pLVX-494 expressed miR-494 effectively (Supplementary Figure 1B). And the stable expression of miR-494 in MDA-231-LUC cells suppressed cell proliferation, colony-forming and cell motility as miR-494 mimics worked (Supplementary Figures 1C–E). MDA-MB-231-LUC cells stably expressing miR-494 (hereafter referred to as pLVX494) were injected into the mammary fat pad of nude mice. We found that overexpression of miR-494 greatly inhibited the tumor-initiating ability of MDA-MB-231-LUC cells. The frequency of primary tumor formed by miR-494-expressing cells was less lower than the control cells (Figure 3a). Moreover, the weight of the tumor enucleated from pLVX-494 group is significantly decreased (Figure 3b). By touching the boundary of the tumor, we found that in 5 of 7 mice primary tumors formed by MDA-231-LUC-pLVX-NC (hereafter referred to as pLVX-NC) invaded into the inside of the peritoneal, whereas all miR-494-expressed tumors were well encased out of the peritoneal (Figure 3c). Consistently, H&E staining showed the pLVX-NC group displayed an obvious tumor invasion into the peritoneal adipose tissue and abdominal muscle tissue, while the pLVX-494 group displayed a sharp demarcation with adjacent adipose or muscle tissue (Figure 3d). Besides detecting the tumorigenesis and invasion *in vivo*, we also explored the miR-494 function in metastasis. 1 × 10^5^ pLVX-494 or pLVX-NC cells were injected into the tail vein of SCID mice, respectively. Measurement of lung metastasis was taken by bioluminescence imaging from the second week. And bioluminescence imaging and lung weight analysis revealed that miR-494 overexpression had decreased the burden of lung metastasis to a certain extent (Figures 3e and f). Meanwhile, H&E staining of lung showed that the metastatic area was also decreased in miR-494-expressing group than that of control group to some extent (Figure 3g). Collectively, these results demonstrated that ectopic miR-494 expression was capable of suppressing tumorigenesis as well as tumor invasion *in vivo.*

### MiR-494 directly targets PAK1 in breast cancer

**Figure 4:**
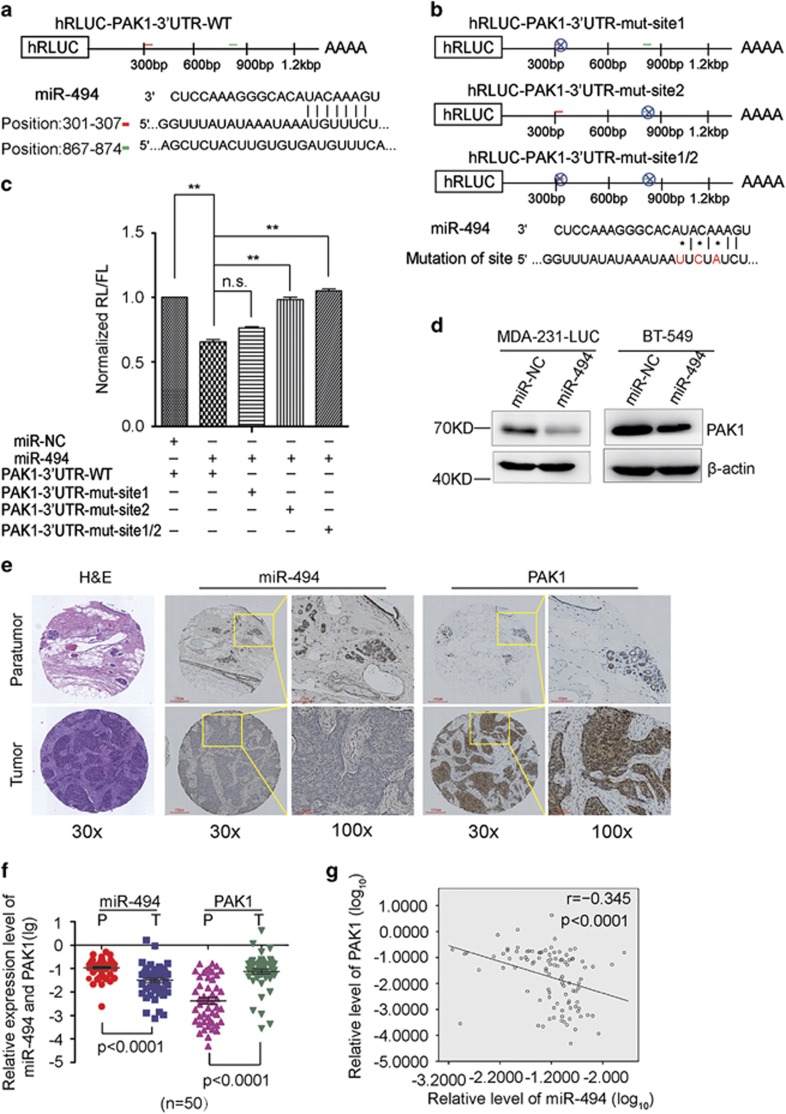
MiR-494 directly targets PAK1 in breast cancer. (**a**) Schematic of predicted miR-494 binding sites in human PAK1-3′UTR. (**b**) Schematic diagram illustrating the mutant site of PAK1-3′UTR. (**c**) The psiCHECK-2 reported plasmids are transiently transfected into HEK293T cells. Luciferase activities are measured after 24 h. (**d**) Western blot assays of PAK1 expression in breast cancer cell lines MDA-231-LUC and BT-549 transfected with miR-NC or miR-494, *β*-actin serves as loading control. (**e**) ISH assays of the expression of PAK1 and miR-494 in breast cancer patient samples. (**f**) The static column of miR-494 (Log10 (mean IOD)) and PAK1 (Log10 (mean IOD)) expression in patient samples (P and T mean adjacent paratumor and paired tumor tissues, respectively). (**g**) The correlation of miR-494 (Log10) and PAK1 (Log10) in clinical breast cancer samples (tissue microarray). Data are presented as mean±S.D. The symbols *, ** and *** represent great significant difference (*P*<0.05, *P*<0.01 and *P*<0.001) by two-tailed Student's *t*-test

It is generally accepted that miRNAs exert their function through regulating the expression of their target genes. To search for putative targets of miR-494, we use bioinformatics prediction software TargetScan (http://targetscan.org) and miRDB (http://mirdb.org) and identified a common set of three candidate genes whose 3′ untranslated regions (3′UTRs) contain at least one putative miR-494 binding sequence and function related with cell proliferation and motility (Supplementary Figure 2). PAK1 is a member of PAK family of serine/threonine kinase, which is amplified in 33% of breast cancer, and has a critical roles in breast cancer.^25^ And also PAK1 serves as targets for the small GTP-binding proteins Cdc42 and Rac,^26^ which is involved in MAPK signaling pathway and actin remodeling. According to Targetscan prediction, there are two miR-494 binding sites on PAK1-3′UTR, site1 (301–307) and site2 (867–874) (Figure 4a). To validate PAK1 as a direct target of miR-494 and find out the exact binding sites, we cloned wild-type and mutant 3′UTR of PAK1 into the downstream of the Renilla luciferase gene respectively in the psiCHECK vector with a firefly luciferase coding gene as internal control (Figure 4b). HEK293T cells were transiently transfected with these constructs together with miR-494 or miR-NC mimics. MiR-494 rather than miR-NC significantly suppressed the luciferase activity of reporter genes containing 3′-UTR of PAK1. Moreover, after transfecting with site2 or site1/2 mutation of PAK1-3′UTR, the suppression of luciferase activity was compromised, but no significance change was observed with site1 transfection. These results indicated that miR-494 directly targeted PAK1 mainly in the position of site2 (Figure 4c). Coherence, ectopic expression of miR-494 markedly reduced PAK1 expression at protein level in MDA-231-LUC and BT-549 cells (Figure 4d). More importantly, we confirmed that the significant downregulation of miR-494 and upregulation of PAK1 in breast cancer tissues compared with paired non-tumor specimens in breast cancer TMA assay (Figure 4e). In addition, the analysis of correlation of PAK1 and miR-494 expression in the TMA showed that the expression of miR-494 and PAK1 was linear negative correlated (Figures 4f and g). Collectively, these data supported that miR-494 directly targeted PAK1 in human breast cancer cells and specimens.

### PAK1 is involved in the biological behaviors of miR-494

**Figure 5:**
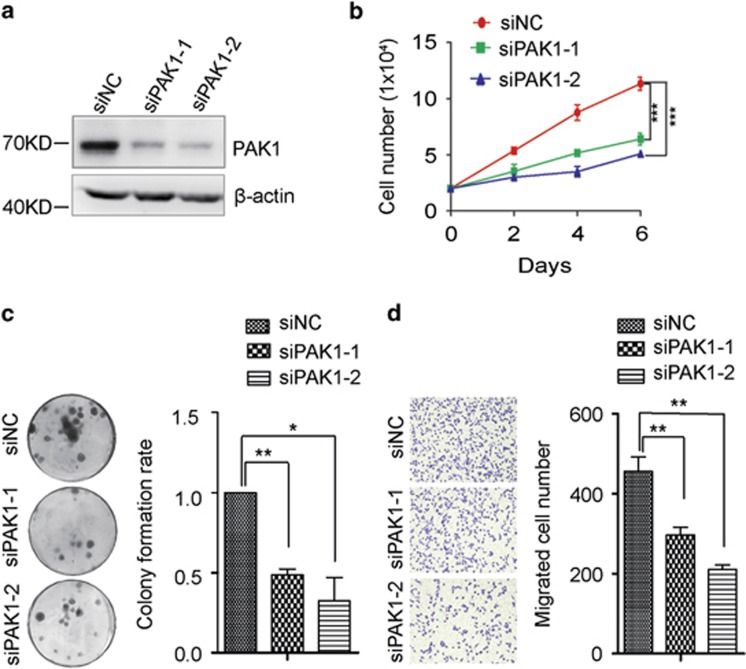
Dysfunction of PAK1 simulates miR-494 mediated biological behaviors in MDA-231-LUC cells. (**a**) Western blot assay shows the efficiency of RNAi against PAK1 in MDA-231-LUC cells, with *β*-actin as loading control. (**b**) Effect of siPAK1 on the proliferation of MDA-231-LUC cells. (**c**) Representative images of colony-forming ability in MDA-231-LUC cells with the transfection of siPAK1. (**d**) Representative images of Transwell migration assay of MDA-231-LUC cells with the transfection of siPAK1. Data are representative of three independent experiments. Bar graphs show means of three experiments±S.D. The symbols *, ** and *** represent great significant difference (*P*<0.05, *P*<0.01 and *P*<0.001) by two-tailed Student's *t*-test

**Figure 6:**
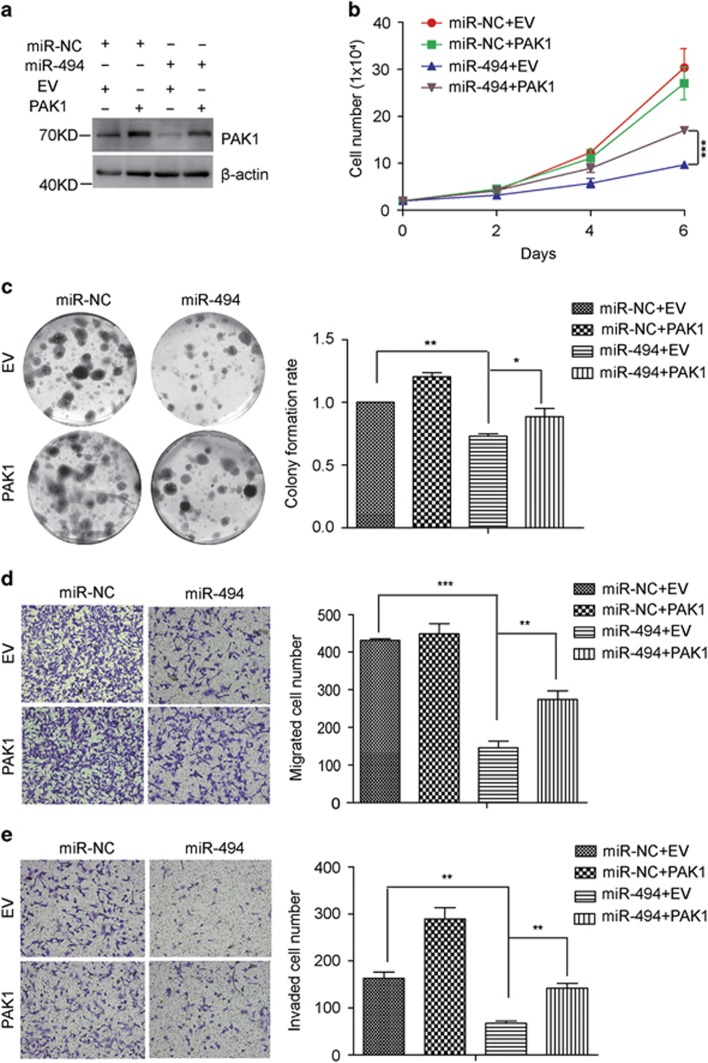
PAK1 re-expression attenuates miR-494 mediated inhibition of cell proliferation, colony formation, migration and invasion in MDA-231-LUC cells. (**a**) Western blot analyzing the expression of PAK1 in MDA-231-LUC cells with the co-transfection of miR-NC or miR-494 together with either pcDNA3.1-EV or pcDNA3.1-PAK1. (**b**) Growth curves of MDA-231-LUC cells with the co-transfection of miR-NC or miR-494 together with either pcDNA3.1-EV or pcDNA3.1-PAK1. (**c**) Colony formation ability assays, (**d**) Transwell migration assays and (**e**) Matrigel invasion assays of the MDA-231-LUC cells with the co-transfection of miR-NC or miR-494 together with either pcDNA3.1-EV or pcDNA3.1-PAK1. Data are representative of three independent experiments. Bar graphs show means of three experiments±S.D. The symbols *, ** and *** represent great significant difference (*P*<0.05, *P*<0.01 and *P*<0.001) by two-tailed Student's *t*-test

To examine the role of PAK1 in breast cancer cell proliferation, colony-formation as well as cell, motility, loss of function assay was performed. Firstly we designed two siRNAs against PAK1 at different sites. And the expression of PAK1 was reduced after siPAK1-1 or siPAK1-2 transfected into MDA-231-LUC and BT-549 cells (Figure 5a; Supplementary Figure 3A). Consistent with previous reports that PAK1 acted as an oncogene in breast cancer,^27^ PAK1 silencing significantly decreased the proliferation ability, colony formation ability and motility in MDA-231-LUC cells (Figures 5b–d), which was similar to the phenotype induced by miR-494. And the same results were observed in BT-549 cells (Supplementary Figure 3). In an attempt to confirm PAK1 was the direct functional mediator of miR-494-induced cell phonotype change, we ectopically expressed PAK1 together with miR-494 in MDA-231-LUC and BT-549 cells. Re-expression of PAK1 was detected at protein level (Figure 6a). As expected, co-transfection of PAK1 with miR-494 profoundly promoted the cell growth and colony forming compared with miR-494 transfection alone (Figures 6b and c). And cell motility suppressed by miR-494 was significantly compromised by the re-expression of PAK1 (Figures 6d and e). The same results were observed in BT-549 cells (Supplementary Figure 4). These findings showed that PAK1 can partially rescue miR-494 induced inhibition of proliferation, colony formation, migration and invasion in MDA-231-LUC cells, which suggested that PAK1 was a functional mediator of miR-494 in breast cancer cells.

## Discussion

miRNAs have recently emerged as salient regulators of cancer processes and are considered as valuable for possible *in vivo* therapeutics.^28^ Here, in this study, we show that miR-494 is downregulated in clinical specimens of breast cancer by both qRT-PCR and *in situ* hybridization of a breast tissue microarray assay. Furthermore, ectopic expression of miR-494 suppresses clonogenic ability and metastasis-relevant traits *in vitro* as well as carcinogenesis and pulmonary metastasis *in vivo*. PAK1, which acts as an oncogene in breast cancer by activating MAPK signal pathway and remolding cytoskeletal, is demonstrated to be a functional target gene of miR-494. These findings strongly suggest that miR-494 has an important role in the initiation and progression of breast cancer.

As well known, miRNAs are highly tissue and cancer type specific, which means one miRNA has different functions in different tissue. Summing up previous reports, we know the expression and function of miR-494 are different among cancers. In lung cancer, miR-494 targets BIM to modulate TRAIL-induced apoptosis.^23^ Although in human cholangiocarcinoma, miR-494 has a global regulatory role in cell cycle progression causing G_2_/M arrest.^29^ In chondrosarcoma, miR-494 targets SOX9 to inhibit cell proliferation and invasion *in vitro.*^30^ In pancreatic cancer, miR-494 is significantly downregulated in pancreatic cancer tissue and is correlated with tumor progression and might be an independent, poor prognostic factor for patient with pancreatic cancer.^31^ From that we see in most of human cancer, miR-494 acts as an antitumor-microRNA. While there are some other studies show that miR-494 activates AKT signal pathway to promote tumor survival and metastasis by targeting PTEN.^32, 33, 34^ In breast cancer, a previous study reported that miR-494 promotes tumor growth and metastasis in 4T1 cell by targeting PTEN.^32^ However, we think that is a special case because PTEN is mutant or lost in most of breast cancer.^35, 36^ Another report suggested that miR-494, together with miR-183, is associated with metastatic events, but miR-494 single has no significance.^37^ To eliminate the error might cause by tumor cells infiltrating in some so called adjacent normal tissue, we perform *in situ* hybridization with a 3′ and 5′ DIG-labeled miR-494 probe on a breast cancer TMA. Under the guide of H&E staining, we can clearly distinguish the tumor and normal tissue. And the result shows miR-494 is thoroughly low expressed in breast cancer tissue compared with normal tissue. And the association between the expression level of miR-494 and breast cancer clinical prognosis is worth further studied.

On the base of the expression difference of miR-494 in breast cancer and normal tissue, we conjecture that miR-494 may have roles in carcinomas biological behaviors. As mentioned before, sustaining proliferative signaling contribute to tumor growth. Growth factor activates MAPK, including extracellular ERKs and JNKs. To investigate whether PAK1 involved in miR-494-mediated function through MAPK signal pathways, we firstly exam the activation of p38, ERK and JNK MAPK signal pathways in miR-494 overexpressed breast cancer cells. We found that only phosphorylation of JNK was downregulated (Supplementary Figure 5A). Furthermore, we assessed the effect of miR-494 on the activation of JNK pathway. Cells were transfected with miR-494 mimics for 48 h and then stimulated with ANS for different time periods. And we found that miR-494 significantly attenuated the phosphorylation of JNK after ANS stimulation (Supplementary Figure 5B). Moreover, we confirmed that the phosphorylation of JNK after ANS stimulation is also compromised after knockingdown of PAK1 expression (Supplementary Figure 5C). To figure out whether JNK pathway is involved in miR-494 and PAK1 mediated biologic functions, we used JNK inhibitor sp600125 to block PAK1-rescued functions. Re-expression of PAK1 in MDA-231-LUC treated with sp600125 was detected by western blot (Supplementary Figure 5D). And as it shows, we can see that PAK1 reverses miR-494-mediated proliferation, migration and invasion suppression, which was jammed by JNK inhibitor sp600125 (Supplementary Figures 5E–G). All these results indicate that JNK pathway, as a downstream of PAK1, is involved in miR-494-mediated biological functions. Consistent with that, there is a reduction of cell proliferation after miR-494 transfection.^38^

F-actin is a component of cytoskeleton and mediate transport in cell.^39^ What is more, cells will form protrusions by polymerizing actin, such as lamellipodia and filopodia and with help of the lamellipodia or filopodia cells realize motility.^40, 41^ In our study, we found that cells transfected with miR-494 mimics show a decrease filament bundle of stress fiber in cytoplasm and an increase of stress fiber bundle assemble in periphery compared with miR-NC (Supplementary Figure 6), which is a kind of actin remodeling type associated with cell motility. Indeed, we find F-actin alteration causes cell migration and invasion ability decreased after the transfection of miR-494. *In vivo* function study, we can see miR-494 significantly inhibits tumorigenesis of breast cancer. Furthermore, we find overexpression of miR-494 decreases breast cancer lung metastasis. To the best of our knowledge, this is the first study to explore the role of miR-494 during malignant progression of breast cancer *in vivo*.

miRNAs acting as oncogenes or tumor suppressor genes is depending on their targets in particular tissue.^42^ PAK1 is an oncogene, which is amplified in several human cancer types, including 30–33% of breast tumor samples and cancer cell lines.^26^ And the amplification of PAK1 is an alternative mechanism for MAPK activation in human breast cancer.^26^ Moreover, PAK1 can change actin cytoskeletal dynamics by activation of LIM-kinase.^43^ PAK1 can also regulate cell lamellipodia spreading,^44^ cell protrusion,^45^ EMT^46^ as well as cell migration.^47^ In our study, we used bioinformatics prediction software of TargetScan and miRDB to predict the potential target genes of miR-494, and finally PAK1 was identified as a functional target gene of miR-494. More importantly, staining of PAK1 and miR-494 in breast cancer samples shows that expression of PAK1 is inversely correlated with that of miR-494. Besides biological targeting, this study shows that PAK1 dysfunction contributes to miR-494 biological functions, especially in cancer progression.

To study the *in vivo* metastasis effect of miR-494 in mice, pLVX-494 or pLVX-NC cells were injected into the tail vein of SCID mice, respectively. The lung metastasis was taken by bioluminescence imaging. The bioluminescence imaging and lung weight analysis revealed that miR-494 overexpression have decreased the burden of lung metastasis to a certain extent. Meanwhile, H&E staining of lung showed that the metastatic area was also decreased in miR-494-expressing group than that of control group to some extent. However, both the decrease of the burden of lung metastasis and the metastatic area are not very strong and seems have no statistical significance. One possible reason is that we injected the miR-494 overexpressing cells into the tail vein of SCID mice instead of implanting the cells into the fat pad of the breast. Thus the initial steps of the metastasis could not be simulated. Tumor cell migration and invasion are key factors in metastatic distribution to distant organs. The initial steps of these processes involve extensive remodeling of the cytoskeleton, disruption of cell adhesions and release of proteases that digest the extracellular matrix. PAK1 plays an important role in regulating these events, as we have shown in Supplementary Figure 6 that cells transfected with miR-494 mimics show an actin remodeling type associated with cell motility, show a decrease filament bundle of stress fiber in cytoplasm and an increase of stress fiber bundle assemble in periphery compared with miR-NC. Thus, to better understand the accurate roles of miR-494 in metastasis cascade of breast cancer, other animal models like implanting the cancer breast cells into the fat pad of the breast and detecting the lung metastasis should be further studied.

All these data clearly shows miR-494 functions as a tumor suppressor in breast cancer. MiR-494 suppresses breast cancer proliferation, colony forming, migration and invasion though PAK1 dysfunction. This study suggests that miR-494 have an important role in tumorigenesis and metastasis and is supposed to be a potential therapeutic target of breast cancer.

## Materials and Methods

### Cell lines and cell culture

Human breast cancer lines BT-474, SK-BR-3 and BT-549 were purchased from the cell bank of the Chinese Academy of Sciences (Shanghai, China). Breast cancer cell line MDA-MB-468, MDA-453, MDA-MB-231, MDA-MB-435S and MCF-10A were provided by Ming-Yao Liu (East China Normal University, Shanghai, China). MCF-7 was obtained from American Type Culture Collection (Manassas, VA, USA) and MDA-MB-231-LUC-D3H2LN was purchased from Perkin Elmer (Alameda, CA, USA). MCF-10A was cultured in DMEM/F12 (GIBCO, Auckland, New Zealand) supplemented with 5% Horse serum (GIBCO), 20 ng/ml EGF (Peprotech, Rocky Hill, NJ, USA), 0.5 mg/ml Hydrocortisone (Stemcell Technology, Vancouver, BC, Canada), 10 *μ*g/ml Insulin (Sigma-Aldrich, St. Louis, MO, USA ), 100 ng/ml Cholera toxin (Sigma-Aldrich). MCF-7 was cultured in DMEM (Hyclone, Logan, UT, USA) supplemented with 10% fetal bovine serum (FBS, GIBCO) and 10 *μ*g/ml insulin. BT-474, SK-BR-3 and BT-549 were cultured in RPMI 1640 (Hyclone) supplemented with 10% FBS. MDA-MB-468, MDA-MB-453, MDA-MB-435S and MDA-MB-231 were cultured in Leibovitz L-15 medium (GIBICO) supplemented with 10% FBS. MDA-MB-231-LUC-D3H2LN was maintained in MEM/EBSS (Hycolne) supplemented with 10% FBS, 1% non-essential amino acids (Hyclone) and 1% sodium pyruvate (Hyclone). HEK-293 T was cultured in DMEM supplemented with 10% FBS. All cell lines were raised at 37 °C in a humidified atmosphere of 5% CO_2_ and 95% air except for MDA-MD-468, MDA-MB-453, MDA-MB-435S and MDA-MB-231, which were raised in a humidified atmosphere containing 100% air.

### Patients and tissue samples

Human breast cancer and corresponding noncancerous tissue used in this study were obtained from patients who underwent surgical resection. A total of 24 paired tissue involved in qPCR assay were from Ruijin Hospital affiliated of Shanghai Jiao Tong University (*n*=16) and First Affiliated of Wenzhou Medical College (*n*=8). These samples were snap-frozen in liquid nitrogen immediately and stored at −80 °C until RNA extraction. Another total of 50 paired tissues used as TMA were provided by Tong-Ji Hospital Affiliated to Tong-Ji University School of Medicine. All patients joined in this study voluntarily with informed consents and this study was performed under the approval of the Research Ethnics Committee of Shanghai Jiao Tong University School of Medicine, the Research Ethnics Committee of Wenzhou Medical College and the Research Ethnic Committee of Tong-Ji University School of Medicine.

### RNA extraction and quantitative real-time PCR

Total RNA tissues and cell lines were extracted from Trizol reagents (Invitrogen, Carlsbad, CA, USA) according to the manufacturer's protocol. cDNA was obtained with ImProm-II Reverse Transcription System (Promega, Madison, WI, USA). Quantitative real-time PCR was performed with SYBR Green PCR Mater Mixture Reagents (Applied Biosystems, Carlsbad, CA, USA) on the ABI 7900HT fast real-time PCR system (Applied Biosystems). Data analysis of miR-494 expression in breast cancer cell line was normalized to the internal control U6 and then evaluated using the 2^ΔΔCt^ method, while data analysis of miR-494 expression in patients tissues was performed using −ΔCt method.

### miRNA *in situ* hybridization

All the breast tissue were embedded in paraffin and breast tissue microarray was synthesized by Shanghai Outdo Biotech (Shanghai, China). *In suit* hybridization was performed with a 3′ and 5′ DIG-labeled miR-494 probe as we described.^23^ ISH slide was scanned by Motic DSAssistant Lite system (Xiamen, China). ISH expression data was analyzed by Image-Pro-Plus. And the data of tumor IODs and areas were collected by Image-Pro Plus 6.0 (Media cybernetic. Inc., Rockville, MD, USA).

### Plasmid construction and transfection

To confirm the possibility of miR-494 targeting predicted candidate gene, 3′UTR of APC, Rab5A and PAK1 containing miR-494 binding site were cloned into the downstream of Renilla luciferase gene in the psiCHECK-2 vector (Promega). Mutant PAK1-3′UTR containing single mutated base and double mutated base sites were constructed using fast mutagenesis kit (Vazyme, shanghai, China). For PAK1 overexpressing, PAK1 was cloned into the pcDNA3.1. And pri-494 was cloned into the lentiviral expression plasmid pLVX-IRES-ZSGreen (Clontech Laboratories, CA, USA) for miR-494 stable overexpressing.

### Western blot

Cells or tissues were lysed with 1 × SDS-lysis buffer, then the total protein was separated by SDS-PAGE and transferred to nitrocellulose membrane (Axygen, Union City, CA, USA). Immunoblotting was performed with a polyclonal antibody against PAK1, JNK, p-JNK, p38, ERK, p-ERK (CST, Beverly, MA, USA). *β*-Actin antibody (CST) was used as an internal loading control. The antigen-antibody complexes were visualized using an ECL detection kit (Millipore, Billerica, MA, USA) and the expression of these proteins were detected with a high sensitive digital imaging equipment (ImageQuant LAS 4000 mini; GE Healthcare Bio-Sciences AB, Uppsala, Sweden). Anisomycin (Selleck, Shanghai, China) short for ANS was used as an activator of JNK. sp600125 (Selleck, Shanghai, China) was used as an inhibitor of JNK.

### *In vivo* tumorigenesis assay

For *in vivo* tumorigenesis assay, 1 × 10^5^ pLVX-NC and pLVX-494 231-LUC cells suspended in 50 *μ*l PBS-containg 25% Matrigel were orthotopically transplanted at the right breast pad of 5-week female nude mice. The tumor incidence was measured 3 weeks post injection and the mice were killed until 6 weeks. And the isolated neoplasm was weighted and then fixed with 4% paraformaldehyde for H&E staining. For *in vivo* pulmonary metastasis assay, 1 × 10^5^ cells were suspended in 200 *μ*l PBS and injected into the lateral tail vein of 5-week female SCID mice. After two weeks, the lung metastasis burden was monitored by detecting the Firefly Luciferase activity, and the measurement was performed at the second week, third week and the fourth week by bioluminescence imaging. The mice were killed at the 4th week and the lung was weighted and then fixed with 4% paraformaldehyde for H&E staining.

### Statistical analysis

All data are presented as the mean±S.D.; groups were compared using two-tailed Student's *t*-test. *P*-values <0.05 were considered significant.
