# Supplementary material for: MicroRNA-494 inhibits breast cancer progression by directly targeting PAK1
Source: Cell Death Dis. 2017 Jan 5;8(1):e2529–. doi: 10.1038/cddis.2016.440 (PMC5386359; doi:10.1038/cddis.2016.440)
Supplement: Supplementary Materials and methods [file cddis2016440x1.docx]

**Supplementary Materials and methods**

1. ***RNA oligonucleotide transfection***

Both negative control (miR-NC) and mature miR-494 were synthesized by Genepharma (Shanghai, China) with the following sequence orientated from 5’ to 3’, miR-494: UGAAACAUACACGGGAAACCUC/GGUUUCCCGUGUAUGUUUCAUU and miR-NC: UUCUCCGAACGUGUCACGUTT/ACGUGACACGUUCGGAGAATT. Two small interfering RNA duple-x oligonucleotides targeting PAK1 mRNA were synthesized by Ruibo (Guangzhou, China),with the following sequence orientated from 5’ to 3’: siPAK1-1: CCAAGAAAGAGCUGAUUAUdTdT/dTdTGGUUCUUUCUCGACUAAUA, siPAK1-2: CAUCCAACAGCCAGAAAUAdTdT/dTdTGUAGGUUGUCGGUCUUUAU. All these RNA oligonucleotide were transfected with Lipofectamine 2000 reagent at concentration of 100nM.

1. ***Cell proliferation and colony formation assays***

Cell growth curve were assayed by seeding 2×10^4^ cells per well in 6-well plates after miR-NC/494 transfection 48hr. Cells dissociation were performed at the 2^nd^, 4^th^ and 6^th^ after seeding and then cell number were counted by Vi-Cell XR Cell Viability Analyzer (Beckman Coulter, CA, USA). To evaluate cell colony forming ability, 500 cells were seeded in 6-well-plates for 14 days to form visible colonies. The colonies were stained with 1% crystal violet (Sigma).

1. ***Cell migration and invasion assays***

Cell migration ability were examined by Corning transwell insert chambers (3422, Corning, NY, USA) and 2×10^4^ cells per well were planted after miR-NC/494 transfection 48hr. Whereas, for invasion ability measurement, 4×10^4^ per well cells were seeded in the matrigel-coated (BD Biosciences, San Jose, CA, USA) transwell insert chamber. And we used 10% FBS as the chemoattractant in the lower well of the chamber. Both MDA-231-LUC and BT-549 were migrated for 18hr. Wound healing assay was implemented by scraping an artificial wound in high confluent cells, and wound closure rate was measured by detecting the closure distance after 24hr.

1. ***F-actin staining***

Cells were planted in the two-well chamber (Thermo Fisher, Waltham, MA, USA) after transfection with miR-NC/494 48hr. 24hr later, cells were fixed with 4% paraformaldehyde for 10 min. After PBS washing twice, cell was permeabilized with formaldehyde for 10 min, and after that 5υl phalloidin (life technologies, Carlsbad, CA, USA) with 200υl 1%BSA was added, incubated for 30min. Photos were verified by confocal scanning laser microscopy (Nikon, Tokyo, Jap).

1. ***Dual luciferase reporter assay***

1x10^5^ HEK-293T cells per well were seeded in 24-well plates at the day before transfection. And then we co-transfected with 100ng of wide type PAK1-3’UTR or mutated PAK1-3’UTR and 100nM miR-NC/494. Cultured at 37℃ for 24hr, luciferase activity was measured with a Dual-Luciferase Reporter System(Promega).

1. ***Western Blot***

Cells or tissues were lysed with 1× SDS-lysis buffer, then the total protein was separated by SDS PAGE and transferred to nitrocellulose membrane (Axygen, Union City, CA, USA). Immunblotting was performed with a polyclonal antibody against PAK1 (CST, Beverly, MA, USA). β-actin antibody (CST) was used as an internal loading control. The antigen-antibody complexes were visualized using an ECL detection kit (Millipore, MA, USA) and the expression of these proteins were detected with a high sensitive digital imaging equipment (ImageQuant LAS 4000 mini; GE Healthcare Bio-Sciences AB, Uppsala).
