# Supplementary material for: MicroRNA-494 inhibits breast cancer progression by directly targeting PAK1
Source: Cell Death Dis. 2017 Jan 5;8(1):e2529–. doi: 10.1038/cddis.2016.440 (PMC5386359; doi:10.1038/cddis.2016.440)
Supplement: Supplementary Information [file cddis2016440x2.doc]

**Legends for Supplemental Figures**

**Figure S1. Function assay of MDA-231-LUC stably transfected with pLVX-494.** (A) Map of the plasmid used to construct miR-494 overexpressed cell line. (B) The efficiency of pLVX-494 overexpressed in MDA-231-LUC. The left is the representative image of the virus transfection efficiency. (C) Growth curve of stable cell lines MDA-231-LUC-pLVX-NC and MDA-231-LUC-pLVX-494. (D) Colony formation of two stable cell lines. (E) Transwell assay of migration ability in the two stable cell lines. Data are representative of three independent experiments. Bar graphs show means of three experiments ± S.D. The symbols *, ** and *** represent great significant difference (p<0.05, p<0.01 and p<0.001) by two-tailed Student’s t-test.

**Figure S2. Candidate target genes of miR-494.**

(A) HEK293T cell was cotransfected with psiCHECK-vector or psiCHECK-APC reporter and miR-494 or miR-NC. (B) HEK293T cell was cotransfected with psiCHECK-vector or psiCHECK-PAK1 reporter and miR-494 or miR-NC. (C) HEK293T cell was cotransfected with psiCHECK-vector or psiCHECK-Rab5A reporter and miR-494 or miR-NC. Data are representative of three independent experiments. Bar graphs show means of three experiments ± S.D. The symbols *, ** and *** represent great significant difference (p<0.05, p<0.01 and p<0.001) by two-tailed Student’s t-test.

**Figure S3. Knockdown of PAK1 imitates miR-494 mediated pathological function of miR-494 in BT-549 cell.**

(A) Western blot assay shows the efficiency of RNAi against PAK1 in BT-549 cell, with β–actin as loading control. (B) Effect of siPAK1 on the proliferation in BT-549 cell. (C) Representative of colony forming ability in BT-549 cell after transfected with siPAK1. (D) Representative images of Transwell migration assay of BT-549 cells after siPAK1 transfection. Data are representative of three independent experiments. Bar graphs show means of three experiments ± S.D. The symbols *, ** and *** represent great significant difference (p<0.05, p<0.01 and p<0.001) by two-tailed Student’s t-test.

**Figure** **S4. PAK1 compromises miR-494 mediate proliferation, colony formation, migration and invasion suppression in BT-549 cell.**

(A) Western blot analyzing the expression of PAK1 in BT-549 cell transfected with miR-NC/494 together with pcDNA3.1-EV/pcDNA3.1-PAK1, with β–actin as loading control. (B) Growth curves of BT-549 cell transfected with miR-NC/494 together with pcDNA3.1-EV/pcDNA3.1-PAK1. (C) Colony formation ability assays for the indicating BT-549 cells transfected with miR-NC/494 together with pcDNA3.1-EV/pcDNA3.1-PAK1. (D) Transwell migration assays measuring migration ability of BT-549 cells transfected with miR-NC/494 together with pcDNA3.1-EV/pcDNA3.1-PAK1. (E) Matrigel invasion assays measuring invasion ability of BT-549 cell transfected with miR-NC/494 together with pcDNA3.1-EV/pcDNA3.1-PAK1. Data are representative of three independent experiments. Bar graphs show means of three experiments ± S.D. The symbols *, ** and *** represent great significant difference (p<0.05, p<0.01 and p<0.001) by two-tailed Student’s t-test.

**Figure S5. JNK pathway is involved in miR-494 and PAK1 mediated cell proliferation, migration and invasion.**

(A) Effects of miR-494 mimics on activation of p38, ERK1/2, c-Jun N-terminal kinase (JNK). (B) MDA-231-LUC cell were transfected with miR-NC or miR-494 mimics and then treated with JNK activator ANS for different time. Cell lysates were analyzed by Western blot using anti-JNK and p-JNK antibody, β-actin was used as the loading control. (C) Western blot analyzing the expression of p-JNK in MDA-231-LUC cell transfected with siNC or siPAK1 and then treated with JNK activator ANS for different time. β-actin was used as the loading control. (D) The expression of PAK1 in MDA-231-LUC cell treated with JNK inhibitor sp600125 and co-transfected with miR-NC or miR-494 together with either pcDNA3.1-EV or pcDNA3.1-PAK1, respectively. β-actin was used as the loading control. (E) PAK1 attenuates miR-494 mediated proliferation inhibition, which can be arrested by JNK inhibitor sp600125. (F) PAK1 compromised miR-494 mediated migration inhibition, which is arrested by JNK inhibitor sp600125. (F) Transwell migration assays and (G) Matrigel invasion assays of the MDA-231-LUC cell transfected with miR-NC or miR-494 mimics together with pcDNA3.1-EV or pcDNA3.1-PAK1 respectively as well as JNK inhibitor sp600125. Data are representative of three independent experiments. Bar graphs show means of three experiments ± S.D. The symbols *, ** represent great significant difference (*p*<0.05, *p*<0.01) by two-tailed Student’s *t*-test.

**Figure S6. F-actin alters after miR-494 transfection.**

Stress fiber in miR-NC transfected cells are mainly in cytoplasm, arranged in a filament, while in miR-494 transfected cells are mainly assembled in cell periphery. The left photos are taken by 40× oil objective, and the bar is 50μm. The right photos are 3 times digital zoom of yellow frame in left photos.
