# Supplementary material for: MicroRNA-494 inhibits breast cancer progression by directly targeting PAK1
Source: Cell Death Dis. 2017 Jan 5;8(1):e2529–. doi: 10.1038/cddis.2016.440 (PMC5386359; doi:10.1038/cddis.2016.440)
Supplement: Supplementary Table S1 [file cddis2016440x3.docx]

Table S1. Characteristics of breast cancer tissues detected by qPCR.
