# Supplementary material for: MicroRNA-494 inhibits breast cancer progression by directly targeting PAK1
Source: Cell Death Dis. 2017 Jan 5;8(1):e2529–. doi: 10.1038/cddis.2016.440 (PMC5386359; doi:10.1038/cddis.2016.440)
Supplement: Supplementary Table S2 [file cddis2016440x4.docx]

Table S2. Characteristics and miR-494 expression in breast cancer tissue detected by qPCR.

Note: ER, estrogen receptor; PR, progesterone receptor; ^a^Independent-Samples T test; ^b^Jonckheere-Terpstra test.
