# Supplementary material for: MicroRNA-494 inhibits breast cancer progression by directly targeting PAK1
Source: Cell Death Dis. 2017 Jan 5;8(1):e2529–. doi: 10.1038/cddis.2016.440 (PMC5386359; doi:10.1038/cddis.2016.440)
Supplement: Supplementary Table S3 [file cddis2016440x5.docx]

Table S3. Characteristics of breast cancer tissues in TMA.

 Table S1. Characteristics of breast cancer tissues in TMA.
