# Supplementary figures and images for: MicroRNA-494 inhibits breast cancer progression by directly targeting PAK1
Source: Cell Death Dis. 2017 Jan 5;8(1):e2529–. doi: 10.1038/cddis.2016.440 (PMC5386359; doi:10.1038/cddis.2016.440)

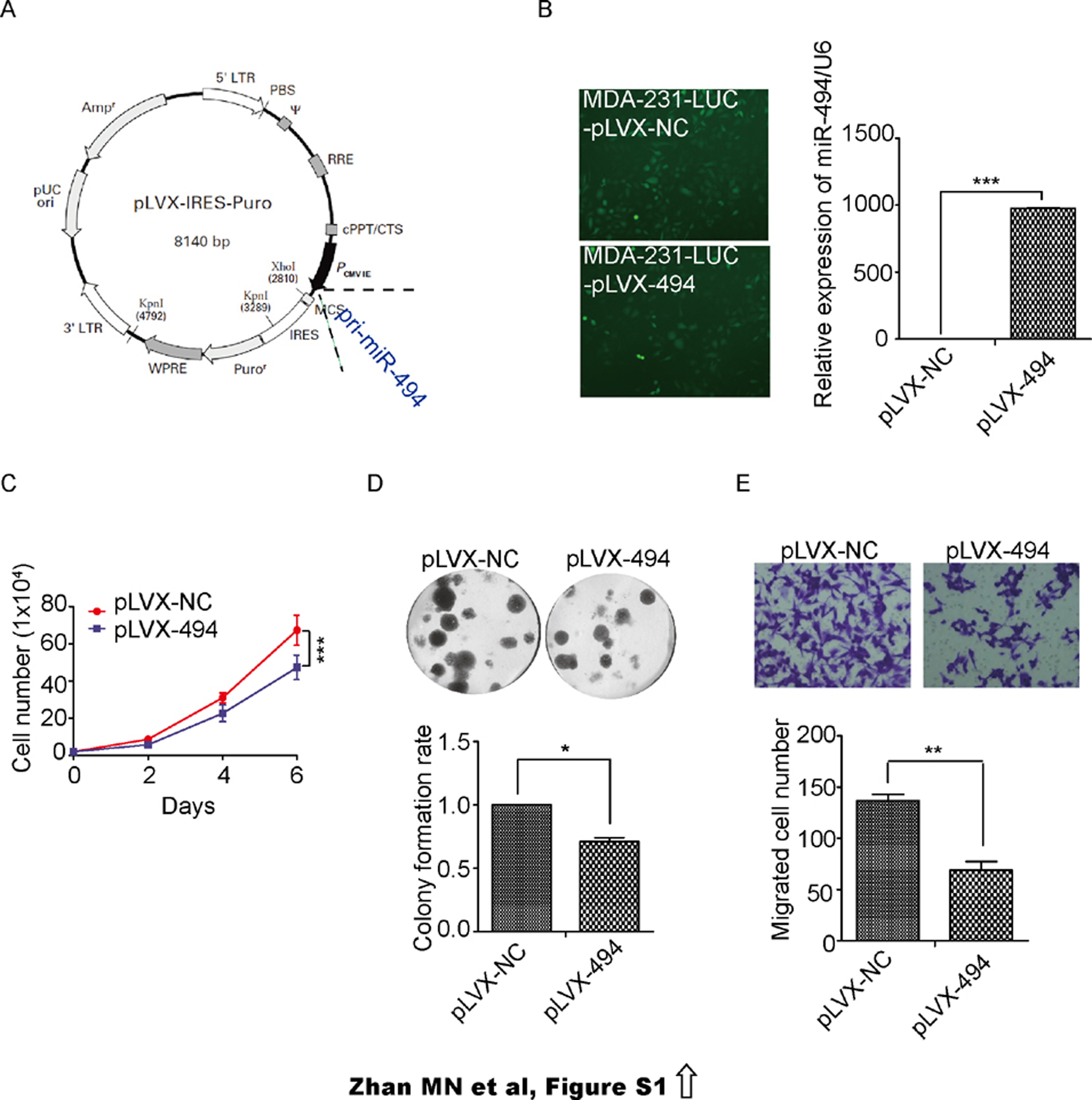

Supplement: Supplementary Figure S1 [file cddis2016440x7.tif]

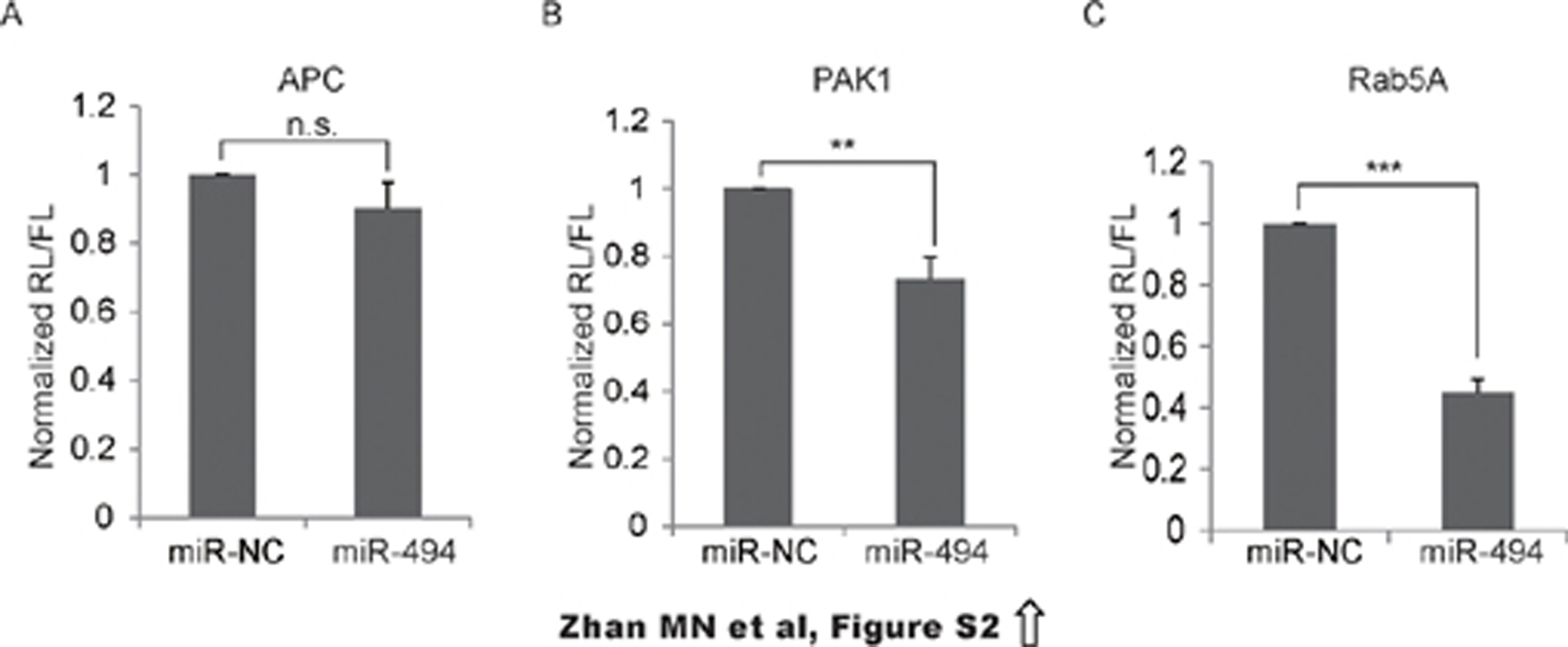

Supplement: Supplementary Figure S2 [file cddis2016440x8.tif]

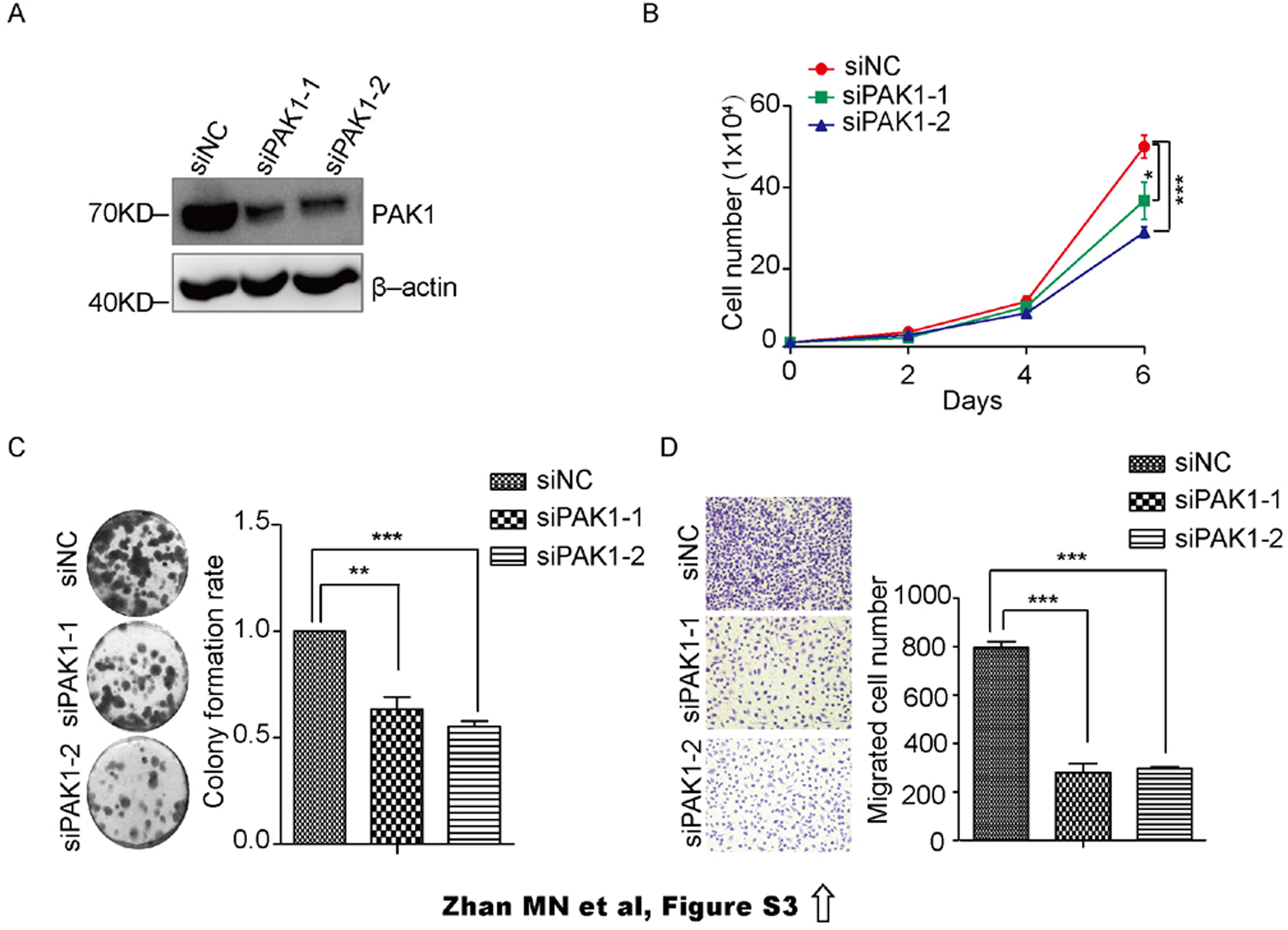

Supplement: Supplementary Figure S3 [file cddis2016440x9.tif]

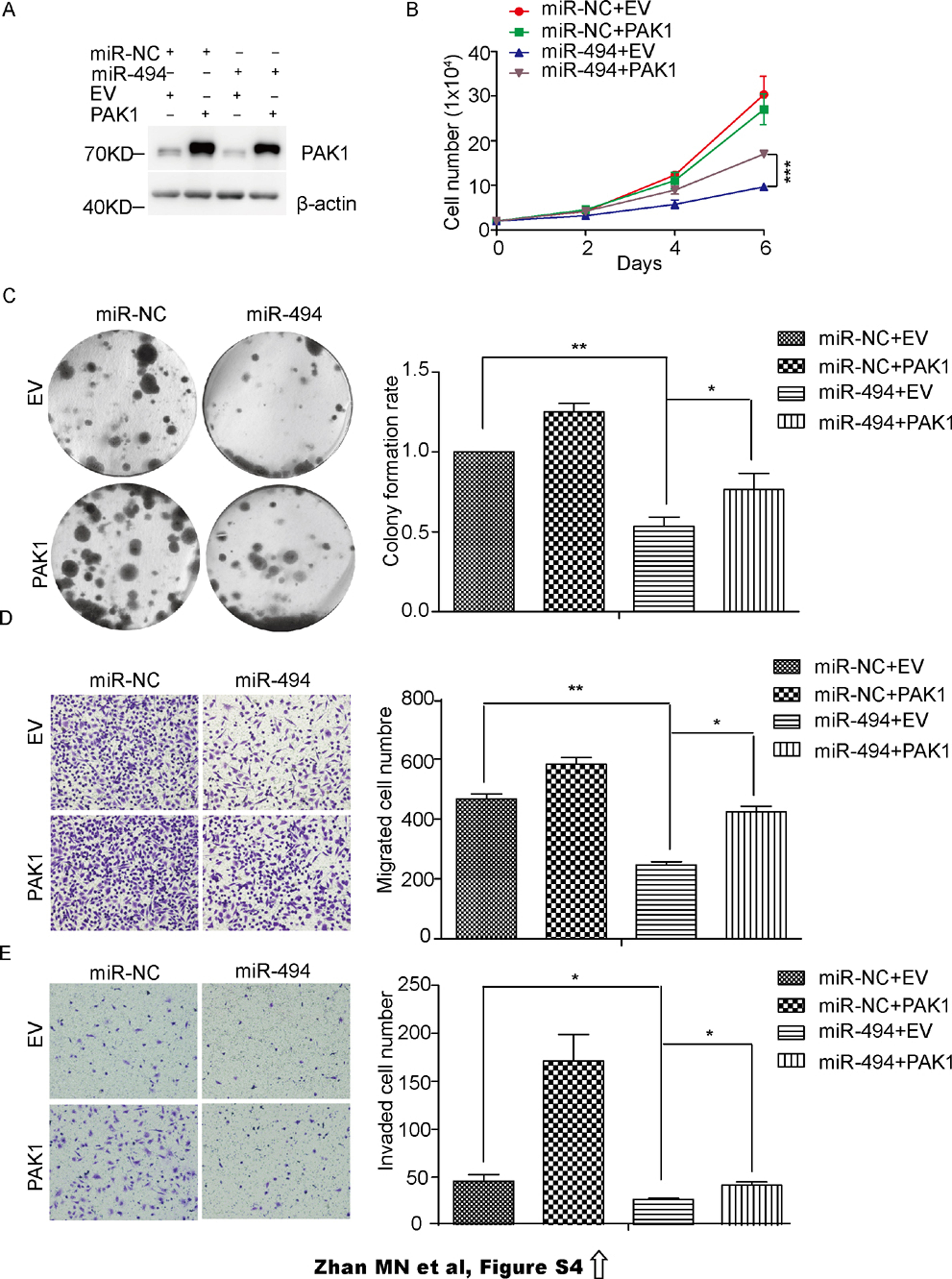

Supplement: Supplementary Figure S4 [file cddis2016440x10.tif]

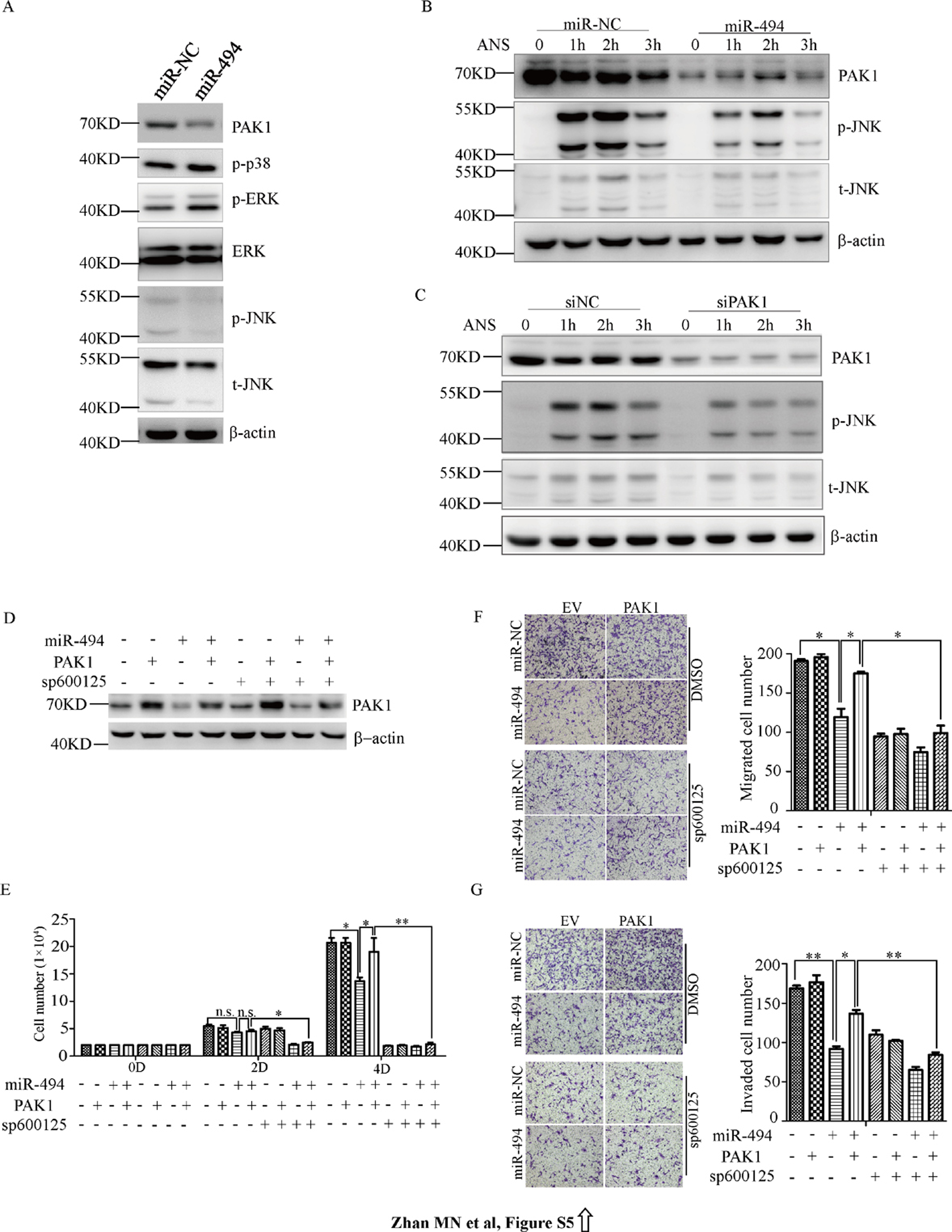

Supplement: Supplementary Figure S5 [file cddis2016440x11.tif]

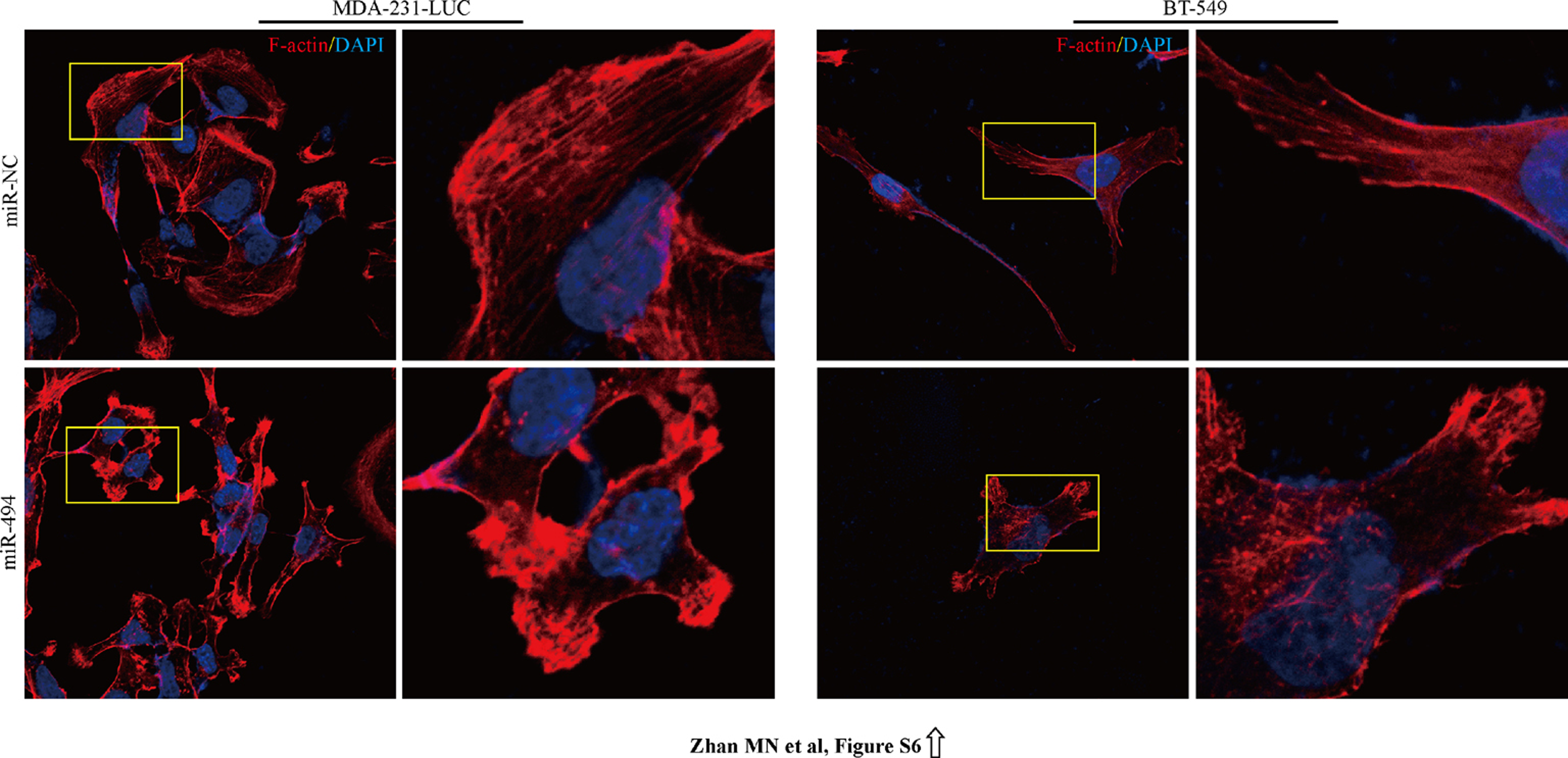

Supplement: Supplementary Figure S6 [file cddis2016440x12.tif]
